# Long non‐coding RNA SNHG16 promotes proliferation and inhibits apoptosis of diffuse large B‐cell lymphoma cells by targeting miR‐497‐5p/PIM1 axis

**DOI:** 10.1111/jcmm.14601

**Published:** 2019-09-04

**Authors:** Qiaojuan Zhu, Yazhao Li, Yang Guo, Linjun Hu, Zunqiang Xiao, Xin Liu, Jiahui Wang, Qiuran Xu, Xiangmin Tong

**Affiliations:** ^1^ Department of Second Clinical Medical College Zhejiang Chinese Medical University Hangzhou China; ^2^ Key Laboratory of Tumor Molecular Diagnosis and Individualized Medicine of Zhejiang Province Zhejiang Provincial People's Hospital (People's Hospital of Hangzhou Medical College) Hangzhou China; ^3^ Center for Translational Medicine The First Affiliated Hospital of Xi'an Jiaotong University Xi'an China; ^4^ Graduate Department BengBu Medical College BengBu China; ^5^ The Medical College of Qingdao University Qingdao China; ^6^ School of Basic Medical Sciences Shandong University Jinan China

**Keywords:** diffuse large B‐cell lymphoma, LncRNA SNHG16, miR‐497‐5p, PIM1, tumour growth

## Abstract

The aberrant expression and dysfunction of long non‐coding RNAs (lncRNAs) have been identified as critical factors governing the initiation and progression of different human cancers, including diffuse large B‐cell lymphoma (DLBCL). LncRNA small nucleolar RNA host gene 16 (SNHG16) has been recognized as a tumour‐promoting factor in various types of cancer. However, the biological role of SNHG16 and its underlying mechanism are still unknown in DLBCL. Here we disclosed that SNHG16 was overexpressed in DLBCL tissues and the derived cell lines. SNHG16 knockdown significantly suppressed cell proliferation and cell cycle progression, and it induced apoptosis of DLBCL cells in vitro. Furthermore, silencing of SNHG16 markedly repressed in vivo growth of OCI‐LY7 cells. Mechanistically, SNHG16 directly interacted with miR‐497‐5p by acting as a competing endogenous RNA (ceRNA) and inversely regulated the abundance of miR‐497‐5p in DLBCL cells. Moreover, the proto‐oncogene proviral integration site for Moloney murine leukaemia virus 1 (PIM1) was identified as a novel direct target of miR‐497‐5p. SNHG16 overexpression rescued miR‐497‐5p‐induced down‐regulation of PIM1 in DLBCL cells. Importantly, restoration of PIM1 expression reversed SNHG16 knockdown‐induced inhibition of proliferation, G0/G1 phase arrest and apoptosis of OCI‐LY7 cells. Our study suggests that the SNHG16/miR‐497‐5p/PIM1 axis may provide promising therapeutic targets for DLBCL progression.

## 1 INTRODUCTION

Diffuse large B‐cell lymphoma (DLBCL) is the most common subtype of non‐Hodgkin's lymphoma. Germinal centre B‐cell‐like (GCB) and activated B‐cell‐like (ABC) are two major molecular subtypes of DLBCL, which are identified by microarray‐based gene expression profiling,^1^ NanoString nCounter system,^2^ immunohistochemistry^3^ and ‘Hans’ classification.^4^ The chromosomal translocation caused by Myc, Bcl‐2 and/or Bcl‐6 structural reorganization is closely related to the therapeutic effect and prognosis of DLBCL.^5^ The standard therapeutic process for patients with DLBCL is rituximab plus cyclophosphamide, doxorubicin, vincristine and prednisone (R‐CHOP). Approximately 60%‐70% of DLBCL patients are cured of disease using this approach. However, approximately 30%‐40% of patients will relapse.^6^ Therefore, it is necessary to better understand the molecular mechanism involved in the initiation and progression of DLBCL and determine new molecular targets as well as therapeutic processes, which will be of great significance for improving the prognosis of DLBCL.

Long non‐coding RNAs (lncRNAs), which are defined as RNA sequence >200 nucleotides in length with no or feeble protein‐coding potential, are critical regulators of physiological and pathological processes including human cancer.^7^ Accumulating studies provide evidence to support the close correlation of the dysregulation of lncRNAs with carcinogenesis, tumour metastasis and therapeutic resistance.^8^, ^9^, ^10^, ^11^, ^12^, ^13^ Mechanistically, lncRNAs modulate biological processes via interacting with other cellular macromolecules, including DNA, RNA and protein, at the transcriptional or post‐transcriptional level.^14^, ^15^, ^16^ Recently, many lncRNAs have been shown to function in cancers by acting as competing endogenous RNAs (ceRNAs), also celebrated as microRNA (miRNA) ‘sponges’ or a miRNA ‘decoy’, which competitively bind to miRNA via the base complementary and thereby increase the abundance of miRNA targets.^17^, ^18^ LncRNA small nucleolar RNA host gene 16 (SNHG16) has been widely investigated in human solid neoplasias.^19^, ^20^, ^21^, ^22^, ^23^ Functionally, SNHG16 promotes cell proliferation, cell cycle progression, migration, invasion and chemotherapy resistance, and it inhibits apoptosis in most human cancers.^19^, ^20^, ^21^, ^22^, ^23^ In contrast, SNHG16 functions as a tumour suppressor in acute lymphoblastic leukaemia (ALL) via repressing cell proliferation and invasion by sponging miR‐124‐3p.^24^ Most studies have revealed that SNHG16 functions as a ceRNA to directly interact with miRNAs and, accordingly, regulate their targets.^21^, ^22^, ^24^ Moreover, SNHG16 acts as an epigenetic regulator to silence p21 and regulates bladder cancer cell proliferation, cell cycle progression and apoptosis.^20^ However, the biological role of SNHG16 and its underlying mechanism in DLBCL are still unknown. SNHG16 acts as a molecular sponge for miR‐497‐5p to promote the proliferation and invasion of papillary thyroid cancer cells.^25^ miR‐497‐5p is underexpressed in human primary NPM‐ALK+ primary lymphoma and inhibits cell cycle progression in cancer cells.^26^ Additionally, miR‐497‐5p functions as a tumour suppressor via targeting the Raf‐1‐mediated MAPK/ERK pathway in multiple myeloma cells.^27^ A previous study has shown that low expression of miR‐497‐5p indicates poor clinical outcomes in DLBCL patients.^28^ Whether SNHG16 exerts a biological function in DLBCL via targeting miR‐497‐5p has not yet been investigated.

In the present study, the expression pattern of SNHG16 was determined in DLBCL tissues and cell lines. Next, in vitro and in vivo experiments were performed to investigate the biological role of SNHG16 and its potential molecular mechanism in DLBCL.

## 2 MATERIALS AND METHODS

### 2.1 Patients and tissue samples

A total of forty‐eight DLBCL tissues (21 GCB and 27 non‐GCB) were collected from patients who underwent surgical resection at Zhejiang Provincial People's Hospital. There were 29 males and 19 females ranging in age from 19 to 78 years old, with a median age of 56 years. All enrolled DLBCL patients had not been previously treated and signed an informed consent to participate in this study. All tumour samples were pathologically confirmed as DLBCL according to the 2008 WHO classification.^29^ Fourteen samples of reactive lymph node hyperplasia (RLH) tissues served as controls. There were eight males and six females ranging in age from 22 to 75 years old, with a median age of 55 years. There was no significant difference in age and sex ratio between the DLBCL group and RLH group. This study was approved by the Ethics Committee of Zhejiang Provincial People's Hospital.

### 2.2 Cell culture

The GCB subtype cell line OCI‐LY7 and ABC subtype cell line OCI‐LY3 were obtained from the ATCC. OCI‐LY7 and OCI‐LY3 cells were incubated in complete Iscove's modified essential medium (IMDM; GIBCO) supplemented with 10% foetal bovine serum (FBS; GIBCO). HEK293T cells were maintained in our laboratory and cultured under standard conditions. The cell culture medium was supplemented with 1% penicillin/streptomycin (Invitrogen). Cell culture was performed at 37°C in an atmosphere containing 5% CO_2_. Normal B lymphocytes were negatively selected from the peripheral blood of a 34‐year‐old healthy female using an immunomagnetic technique (Human B Cell Enrichment Kit, Stem Cell Technologies).

### 2.3 Transfection and lentiviral vector infection

miR‐497‐5p mimics, miR‐497‐5p inhibitors (anti‐miR‐497‐5p) and control clones were purchased from RiboBio. The cDNA encoding SNHG16 was PCR‐amplified by the Pfu Ultra II Fusion HS DNA Polymerase (Stratagene, Agilent Technologies), and the full‐length proviral integration site for the Moloney murine leukaemia virus 1 (PIM‐1) ORF was obtained from human mRNA by reverse transcription‐PCR (RT‐PCR). The clone sequences were controlled for artificial point mutations and inserted into pcDNA3.1 (Invitrogen). These plasmids were transfected into cells using Lipofectamine 2000 (Invitrogen) according to the manufacturer's instructions. The lentivector‐mediated SNHG16 shRNAs (SNHG16 shRNA‐1 and SNHG16 shRNA‐2) and non‐targeting (NT) shRNA were obtained from RiboBio. Polybrene (8 μg/mL; Sigma‐Aldrich) was added to the culture medium to increase the efficiency of infection.

### 2.4 Western blot analysis

Total protein was extracted using RIPA lysis buffer (Beyotime). A BCA kit (Pierce) was used to measure the protein concentration. Equal amounts of protein samples (20 μg) were subjected to 10% SDS‐PAGE and transferred onto polyvinylidene fluoride (PVDF) membranes (Millipore). The membranes were incubated with primary antibodies, which were diluted in 5% TBST skimmed milk, overnight at 4°C and subsequently incubated with horseradish peroxidase (HRP)‐conjugated antimouse or anti‐rabbit IgG at room temperature for 1‐2 hours. Then, the membranes were visualized with ECL reagents (Millipore) and imaged using an Amersham Imager 600 from GE Healthcare Life Sciences. PIM1 antibody (sc‐13513), proliferating cell nuclear antigen (PCNA) antibody (sc‐25280), Bcl‐2 antibody (sc‐7382) and β‐actin antibody (sc‐8432) were purchased from Santa Cruz Biotechnology.

### 2.5 RNA extraction and quantitative real‐time PCR (qRT‐PCR)

Total RNA was extracted using TRIzol reagent (Invitrogen) according to the manufacturer's procedure and reverse‐transcribed into cDNA using a PrimeScript reverse transcriptase reagent kit (Takara) or TaqMan MicroRNA Reverse Transcription kit (Applied Biosystems) according to the manufacturer's instructions. Next, qRT‐PCR was conducted with the Applied Biosystems 7500 Sequence Detection system using a miRNA‐specific TaqMan miRNA Assay Kit (Applied Biosystems) and the SYBR Premix Ex Taq™ Kit (Takara). The primers are listed in Table 1.

**Table 1:** Primers used in qRT‐PCR analysis

Name		Sequence (5′ → 3′)
miR‐497‐5p	RT primer	GTCGTATCCAGTGCAGGGTCCGAGGTATT CGCACTGGATACGACACAAAC	
	Forward	GTGCAGGGTCCGAGGT	
	Reverse	TAGCCTGCAGCACACTGTGGT	
U6	RT primer	GAACGCTTCACGAATTTGCGTGTCAT	
	Forward	CTCGCTTCGGCAGCACA	
	Reverse	AACGCTTCACGAATTTGCGT	
SNHG16	Forward	CCCAAGCTTGCGTTCTTTTCGAGGTCGGC	
	Reverse	CCGGAATTCTGACGGTAGTTTCCCAAGTT	
PIM1	Forward	GAGAAGGACCGGATTTCCGAC	
	Reverse	CAGTCCAGGAGCCTAATGACG	
GAPDH	Forward	AGGTCGGTGTGAACGGATTTG	
	Reverse	TGTAGACCATGTAGTTGAGGTCA	

### 2.6 Cell proliferation and flow cytometry analysis

Cell proliferation was detected via the CCK‐8 (Cell Counting Kit‐8, Dojindo) assay as previously described.^13^ The cell cycle distribution and apoptosis were measured using a FACSCanto II flow cytometer (BD Biosciences). PI/RNase Staining Buffer (BD Biosciences) and the PE Annexin V Apoptosis Detection Kit I (BD Biosciences) were used for these assays as previously described.^13^

### 2.7 In vivo tumour xenograft experiments

The animal studies were approved by the Institutional Animal Care and Use Committee of Xi'an Jiaotong University, Xi'an, China. Eight male NOD/SCID mice (6‐8 weeks old) were raised in an SPF environment in the animal facility during the experimental procedures. Briefly, low‐passage 5 × 10^6^ OCI‐LY7 cells with or without SNHG16 knockdown that were suspended in 100 μL PBS were subcutaneously injected into the left flank of mice. Tumour volumes were monitored every 3 days after tumour appearance using an electronic digital caliper. The tumour volume was calculated as follows: (smallest diameter^2^ × largest diameter)/2.^30^ After 3 weeks, the mice were killed, and the xenograft tumour tissues were analysed by qRT‐PCR and immunoblotting for SNHG16, PCNA and Bcl‐2 expression.

### 2.8 Luciferase reporter assay

The pEZX‐MT06 luciferase reporter vector containing the 3′UTR of PIM1 was obtained from GeneCopoeia Inc. The synthetic SNHG16 fragment was subcloned into the pGL3 luciferase reporter vector (Promega). The potential binding sites of miR‐497‐5p in the 3′UTR of PIM1 and SNHG16 were mutated using a Quick‐change site‐directed mutagenesis kit (Agilent Technologies). HEK293T cells were cotransfected with different luciferase reporter vectors and miR‐497‐5p mimics or inhibitors. Forty‐eight hours after transfection, luciferase activity was measured with the dual‐luciferase assay system (Promega).

### 2.9 Pull‐down assay with biotinylated miR‐497‐5p

OCI‐LY7 cells were transduced with biotin‐labelled wild‐type (wt) miR‐497‐5p and mutated (mt) miR‐497‐5p plasmids (RiboBio), respectively. Forty‐eight hours after transfection, cells were harvested and incubated with a specific lysate buffer (Ambion) for 10 minutes. Then, the cell lysates were mixed with M‐280 streptavidin magnetic beads (Sigma) for 3 hours at 4°C. A biotin‐labelled antagonistic miR‐497‐5p probe was used as a negative control (Bio‐NC). The pull‐down products were subjected to qRT‐PCR to evaluate SNHG16 expression.

### 2.10 Statistical analysis

Data analysis was performed using GraphPad Prism 6.0 Software (GraphPad Inc). All data are presented as the mean ± SD. Differences between groups were analysed by Student's *t* test or ANOVA. The correlation analysis was performed by Pearson's correlation test. *P* < .05 was considered to indicate statistical significance.

## 3 RESULTS

### 3.1 SNHG16 is overexpressed in DLBCL

**Figure 1:**
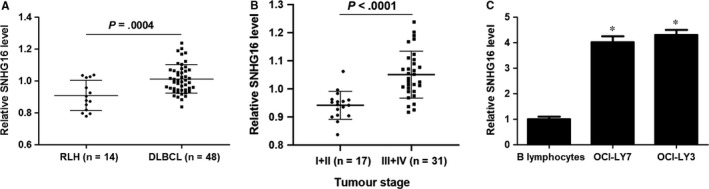
SNHG16 is up‐regulated in DLBCL. A, The relative levels of SNHG16 in forty‐eight samples of DLBCL tissues and fourteen cases of RLH tissues are shown. B, DLBCL tissues from patients with advanced tumour stages (n = 31) showed prominently higher levels of SNHG16 compared with those from patients with early tumour stages (n = 17). C, The levels of SNHG16 between DLBCL cell lines (OCI‐LY7 and OCI‐LY3) and B lymphocytes were analysed by qRT‐PCR. n = three independent repeats, *P < .05

We detected the expression of SNHG16 in DLBCL tissues and RLH tissues using qRT‐PCR. As shown in Figure 1A, the expression of SNHG16 was significantly up‐regulated in DLBCL compared with RLH tissues (*P* = .0004). Moreover, DLBCL tissues from patients with advanced tumour stages showed prominently higher levels of SNHG16 compared with those from patients with early tumour stages (*P* < .0001, Figure 1B). Next, we further determined the levels of SNHG16 in B lymphocytes and DLBCL cell lines. Consistently, the expression of SNHG16 in both OCI‐LY7 and OCI‐LY3 cells was prominently higher than that in B lymphocytes (*P* < .05, Figure 1C). Thus, SNHG16 was highly expressed in DLBCL.

### 3.2 SNHG16 regulates DLBCL cell proliferation, cell cycle progression and apoptosis

**Figure 2:**
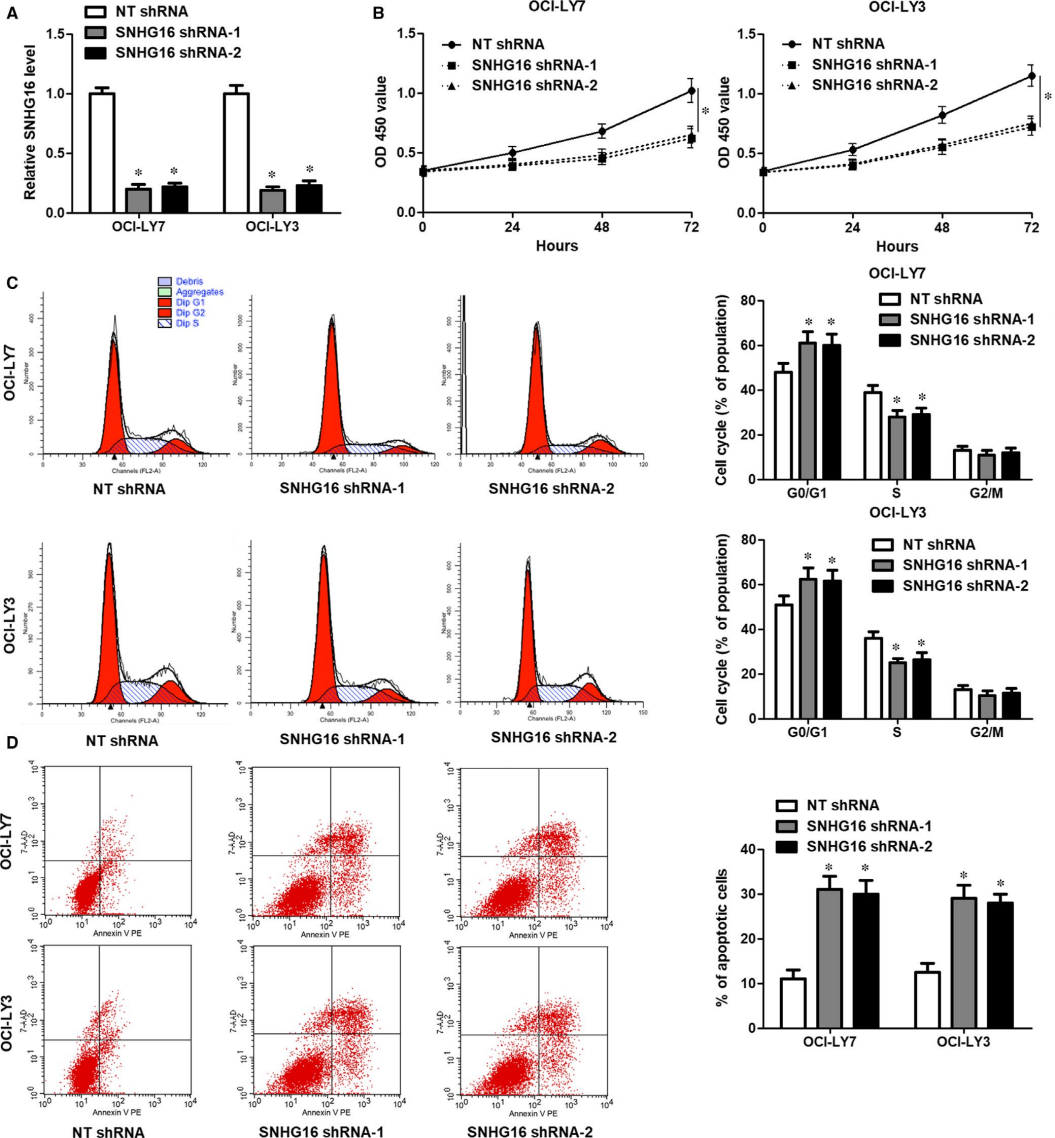
Knockdown of SNHG16 represses DLBCL cell proliferation and cell cycle progression, and induces apoptosis. A, Lentivector‐mediated SNHG16 shRNAs (SNHG16 shRNA‐1 and SNHG16 shRNA‐2) or non‐targeting (NT) shRNA were transduced into OCI‐LY7 and OCI‐LY3 cells, and qRT‐PCR was subsequently performed to determine SNHG16 expression. B, CCK‐8 assays found that SNHG16 knockdown repressed the proliferation of DLBCL cells. C, Knockdown of SNHG16 resulted in cell cycle arrest at G0/G1 phase in both OCI‐LY7 and OCI‐LY3 cells. D, The percentage of apoptotic DLBCL cells was significantly elevated by SNHG16 knockdown. n = three independent repeats, *P < .05

As SNHG16 was aberrantly overexpressed in DLBCL, we further investigated its biological role. We markedly down‐regulated SNHG16 expression by transfecting different shRNAs into two DLBCL cell lines, OCI‐LY7 and OCI‐LY3 cells (*P* < .05, Figure 2A). The CCK‐8 assay showed that SNHG16 knockdown prominently reduced the proliferation of DLBCL cells (*P* < .05, Figure 2B). Additionally, knockdown of SNHG16 led to cell cycle arrest at G0/G1 phase in both OCI‐LY7 and OCI‐LY3 cells (*P* < .05, Figure 2C). Furthermore, the portion of apoptotic DLBCL cells was obviously increased by SNHG16 knockdown (*P* < .05, Figure 2D). Taken together, SNHG16 knockdown inhibited cell proliferation and cell cycle progression, and it induced apoptosis of DLBCL cells.

### 3.3 Knockdown of SNHG16 suppresses in vivo growth of DLBCL cells

**Figure 3:**
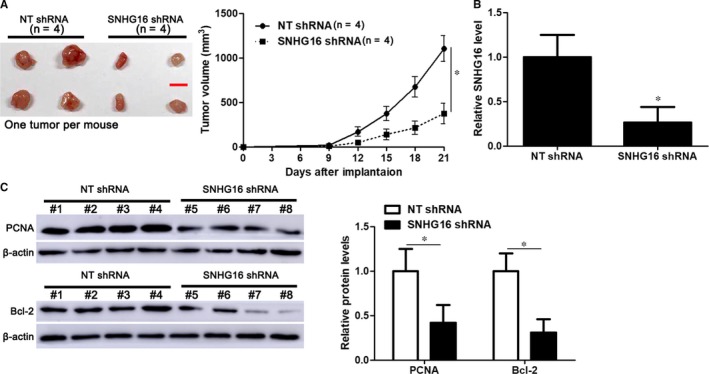
SNHG16 knockdown inhibits in vivo tumour growth of DLBCL cells. A, OCI‐LY7 cells that were transfected with lentivector‐mediated SNHG16 shRNA or non‐targeting (NT) shRNA were subcutaneously implanted into NOD/SCID mice. The volume of tumours initiated by OCI‐LY7 cells with SNHG16 knockdown (n = 4) was significantly reduced compared with the control group (n = 4). B, The expression of SNHG16 was markedly lower in xenograft tumour tissues collected from SNHG16 knockdown group (n = 4) than the control group (n = 4). C, Immunoblotting analysis indicated that the levels of PCNA and Bcl‐2 protein were significantly lower in xenograft tumour tissues collected from the SNHG16 knockdown group (n = 4) than the control group (n = 4). *P < .05

Next, the oncogenic role of SNHG16 was further confirmed in vivo. OCI‐LY7 cells with or without SNHG16 were subcutaneously injected into mice. Tumour growth curves suggested that SNHG16 knockdown markedly inhibited in vivo tumour growth of OCI‐LY7 cells (*P* < .05, Figure 3A). The expression of SNHG16 in xenograft tumour tissues collected from the SNHG16 knockdown group was significantly lower than in the control group (*P* < .05, Figure 3B). Moreover, immunoblotting analysis was performed to detect the levels of the proliferation (PCNA) marker and anti‐apoptosis marker (Bcl‐2) in xenograft tumour tissues. Accordingly, the levels of PCNA and Bcl‐2 were significantly lower in xenograft tumour tissues collected from the SNHG16 knockdown than the control group (*P* < .05, Figure 3C). Altogether, SNHG16 exerted a tumour‐promoting role in DLBCL.

### 3.4 SNHG16 functions as a ceRNA by interacting with miR‐497‐5p

**Figure 4:**
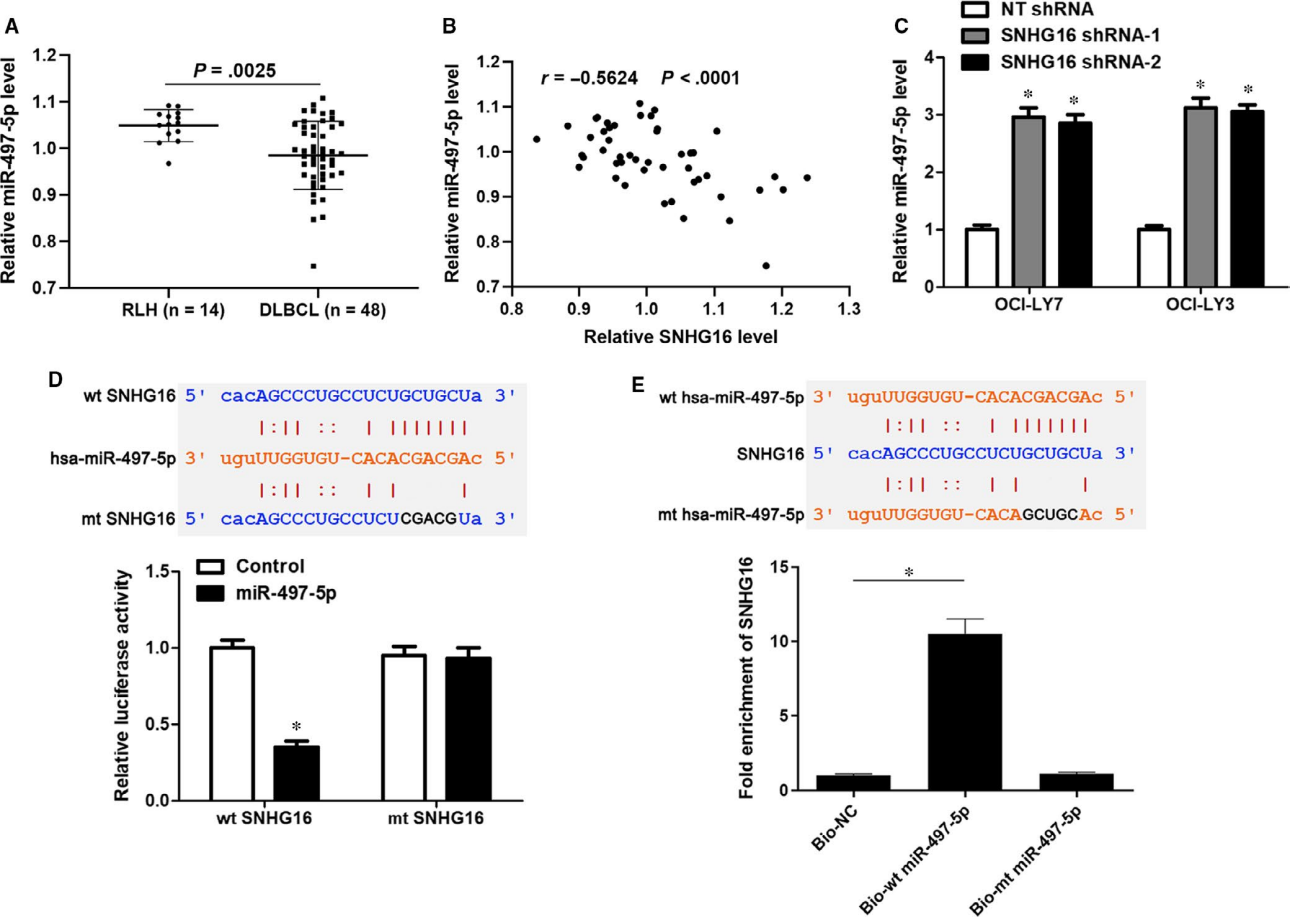
SNHG16 functions as a ceRNA by sponging miR‐497‐5p. A, The relative levels of miR‐497‐5p in forty‐eight samples of DLBCL tissues and fourteen cases of RLH tissues are shown. B, The expression of SNHG16 was inversely correlated with the miR‐497‐5p level in DLBCL tissues (n = 48). C, Lentivector‐mediated SNHG16 shRNAs (SNHG16 shRNA‐1 and SNHG16 shRNA‐2) or non‐targeting (NT) shRNA were transduced into OCI‐LY7 and OCI‐LY3 cells, and qRT‐PCR was subsequently performed to evaluate miR‐497‐5p expression. D, Luciferase reporter vectors containing wild‐type (wt) or mutated (mt) SNHG16 and miR‐497‐5p mimics or negative control were cotransfected into HEK293T cells, and the relative luciferase activity was detected. E, SNHG16 was pulled down with biotinylated wt miR‐497‐5p. However, there was no interaction between SNHG16 and mt miR‐497‐5p. n = three independent repeats, *P < .05

Subsequently, we attempted to investigate the molecular mechanism involved in the biological role of SNHG16 in DLBCL. According to the starBase V3.0 online platform,^31^, ^32^ more than a dozen candidate target miRNAs for SNHG16 were predicted. Only miR‐497‐5p was previously indicated as a candidate tumour suppressor in DLBCL^28^ and inversely correlated with SNHG16 level in DLBCL samples from the TCGA database (*r* = −.422, *P* = .0031, Figure S1A). qRT‐PCR data revealed that miR‐497‐5p expression in DLBCL tissues was significantly lower than in RLH tissues (*P* = .0025, Figure 4A). Moreover, an inverse correlation was observed between SNHG16 and miR‐497‐5p expression in both DLBCL and RLH tissues (*P* < .05, Figure 4B and Figure S1B). Next, SNHG16 knockdown marked increased levels of miR‐497‐5p in both OCI‐LY7 and OCI‐LY3 cells (*P* < .05, Figure 4C). As shown in Figure 4D, miR‐497‐5p overexpression decreased the luciferase activity of vectors containing wt SNHG16 (*P* < .05), whereas the fluorescence intensity of vectors containing mt SNHG16 was not changed after miR‐497‐5p overexpression. Moreover, SNHG16 was obviously pulled down by biotinylated wt miR‐497‐5p (*P* < .05, Figure 4E). However, mutagenesis of the binding sites between SNHG16 and miR‐497‐5p abolished this interaction (Figure 4E). Thus, the above results indicated that SNHG16 directly interacted with miR‐497‐5p and regulated its abundance in DLBCL cells.

### 3.5 PIM1 is a direct target of miR‐497‐5p

**Figure 5:**
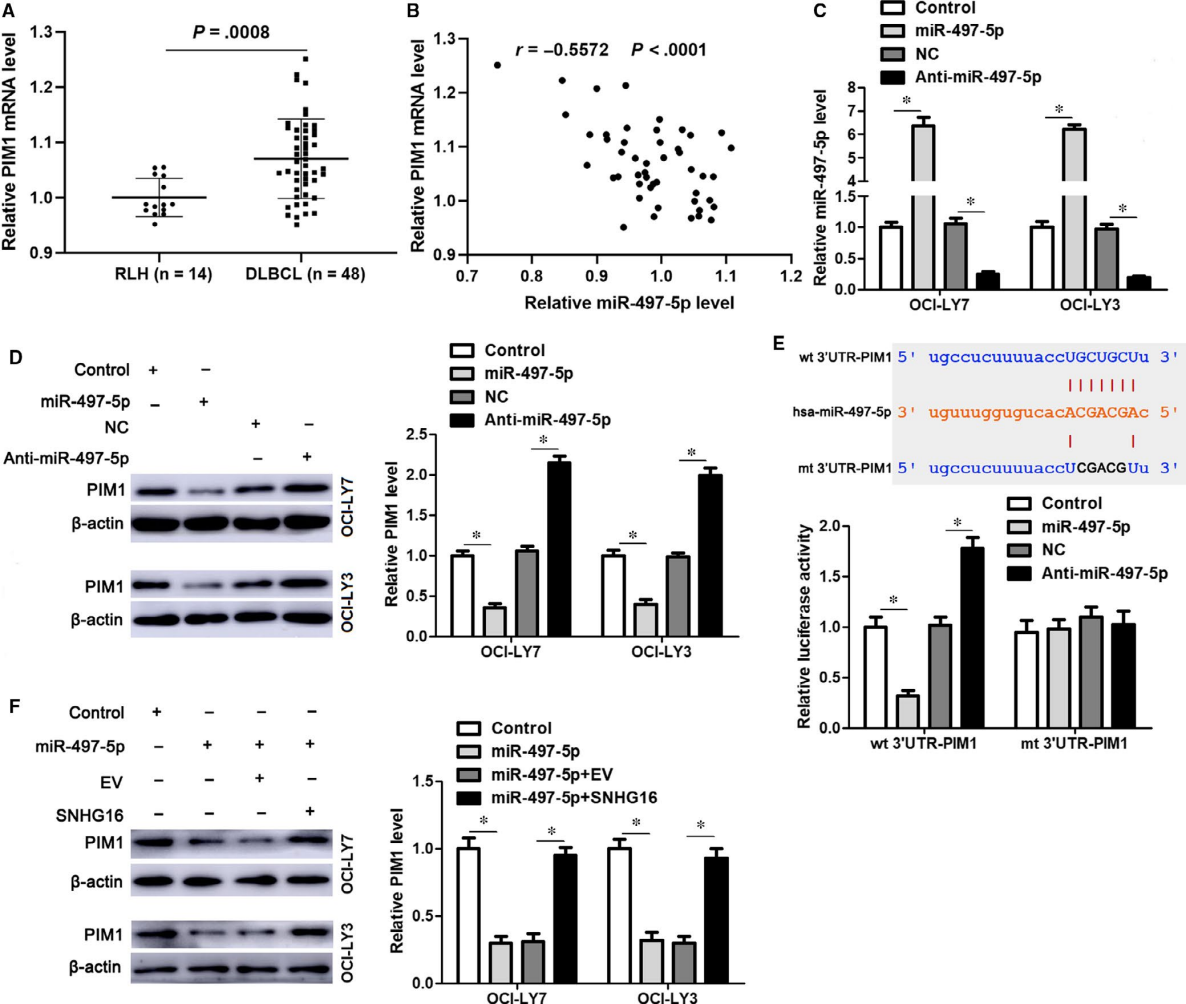
PIM1 is identified as a novel target of miR‐497‐5p. A, The relative levels of PIM1 mRNA in forty‐eight samples of DLBCL tissues and fourteen cases of RLH tissues are shown. B, The expression of PIM1 mRNA was inversely correlated with the miR‐497‐5p level in DLBCL tissues (n = 48). C, miR‐497‐5p mimics or inhibitors (anti‐miR‐497‐5p) and corresponding control clones were transfected into OCI‐LY7 and OCI‐LY3 cells, and qRT‐PCR was subsequently performed to evaluate miR‐497‐5p expression. D, miR‐497‐5p overexpression reduced whereas miR‐497‐5p knockdown increased the expression of PIM1 protein in both OCI‐LY7 and OCI‐LY3 cells. E, Luciferase reporter vectors containing wild‐type (wt) or mutated (mt) 3′UTR of PIM1 and miR‐497‐5p mimics or inhibitors were cotransfected into HEK293T cells, and the relative fluorescence intensity was detected. F, OCI‐LY7 and OCI‐LY3 cells were transfected with corresponding vectors. SNHG16 overexpression rescued miR‐497‐5p‐induced repression of PIM1 in DLBCL cells. n = three independent repeats, *P < .05

The starBase V3.0 platform suggested dozens of targets for miR‐497‐5p. The proto‐oncogene PIM1, which was negatively correlated with miR‐497‐5p expression in DLBCL tissues (*r* = −.543, *P* < .0001, Figure S1C) and acted as an oncogene in DLBCL,^33^ was predicted as a candidate target of miR‐497‐5p. The qRT‐PCR data revealed significantly elevated expression of PIM1 mRNA in DLBCL tissues than in RLH tissues (*P* = .0008, Figure 5A). Moreover, an inverse correlation between PIM1 mRNA and miR‐497‐5p expression was observed in both DLBCL and RLH tissues (*P* < .05, Figure 5B and Figure S1D). Notably, miR‐497‐5p overexpression markedly reduced the levels of PIM1 protein, whereas miR‐497‐5p knockdown prominently led to increased expression of PIM1 protein in both OCI‐LY7 and OCI‐LY3 cells (*P* < .05, Figure 5C,D). Importantly, miR‐497‐5p overexpression reduced whereas miR‐497‐5p knockdown increased the fluorescence intensity of vectors containing the wt 3′UTR of PIM1 (*P* < .05, Figure 5E). However, modulation of the miR‐497‐5p level had no significant impact on the luciferase activity of vectors containing the mt 3′UTR of PIM1 (Figure 5E). Interestingly, SNHG16 overexpression rescued miR‐497‐5p‐induced down‐regulation of PIM1 in both OCI‐LY7 and OCI‐LY3 cells (*P* < .05, Figure 5F). Thus, SNHG16 functioned as a ceRNA to sponge miR‐497‐5p and subsequently regulate PIM1 expression.

### 3.6 PIM1 restoration reverses SNHG16 knockdown‐attenuated DLBCL cell growth

**Figure 6:**
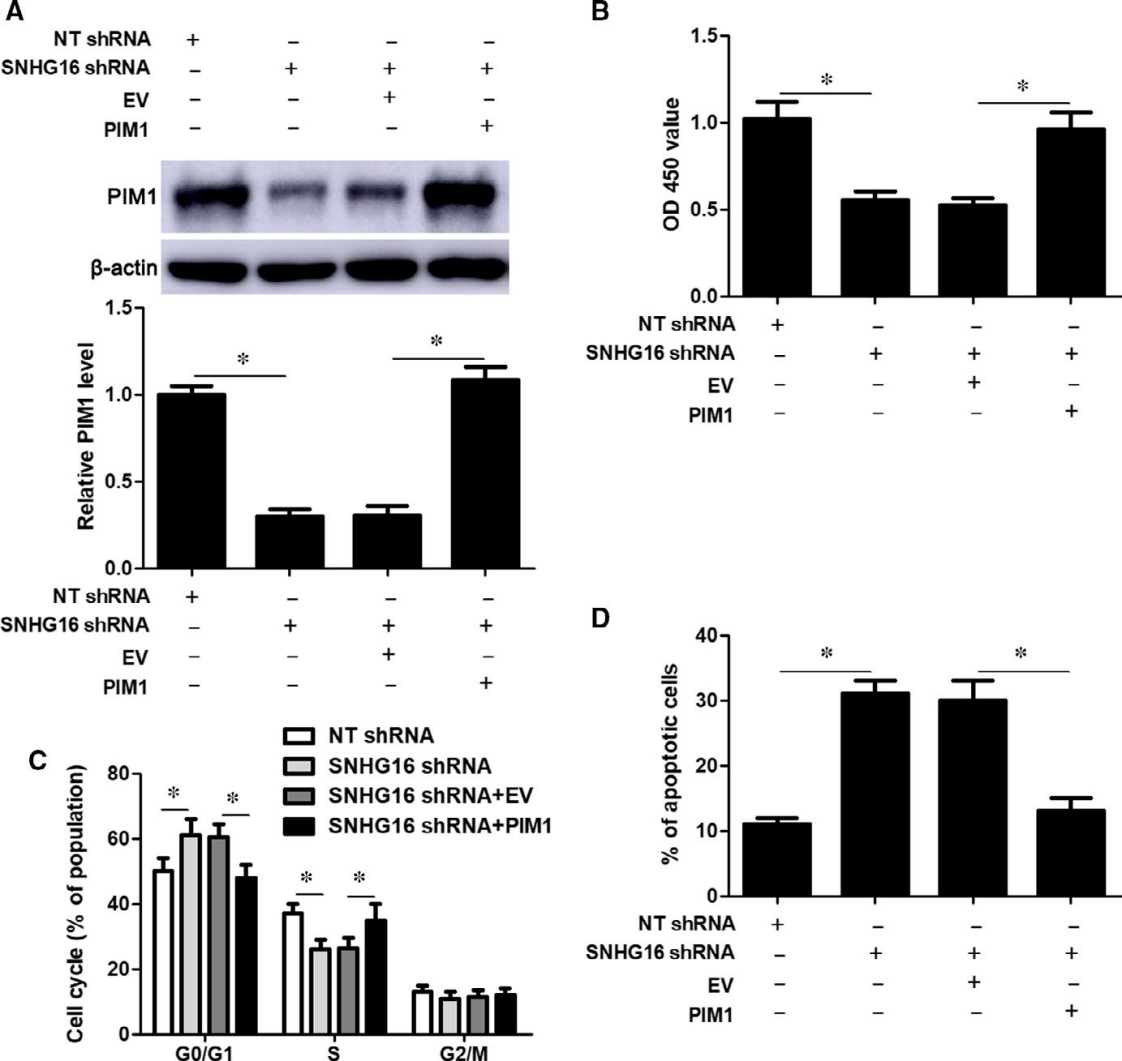
PIM1 restoration reverses SNHG16 knockdown‐induced growth arrest of OCI‐LY7 cells. A, PIM1 expression was restored by transfecting expression plasmids into OCI‐LY7 cells with SNHG16 knockdown, and immunoblotting was performed to detect PIM1 expression. B, CCK‐8, (C) flow cytometry‐based apoptosis detection and (D) cell cycle distribution analysis were performed to evaluate the proliferation, apoptosis and cell cycle progression of OCI‐LY7 cells after transfection with the corresponding vectors. n = three independent repeats, *P < .05. EV, empty vector

Rescue experiments were performed to disclose whether PIM1 was a downstream effector of SNHG16 in DLBCL cells. The expression of PIM1 was restored by transfecting expression plasmid into OCI‐LY7 cells with SNHG16 knockdown (*P* < .05, Figure 6A). PIM1 restoration rescued SNHG16 knockdown‐induced inhibition of proliferation and G0/G1 phase arrest in OCI‐LY7 cells (*P* < .05, Figure 6B,C). Furthermore, PIM1 overexpression reduced apoptosis of OCI‐LY7 cells with SNHG16 knockdown (*P* < .05, Figure 6D). Taken together, SNHG16 functioned as an oncogene by targeting the miR‐497‐5p/PIM1 axis in DLBCL cells.

## 4 DISCUSSION

Previous studies have reported the aberrant expression of lncRNAs in DLBCL.^34^, ^35^ However, the expression pattern of SNHG16 in DLBCL has remained unexplored. In the present study, we found that increased expression of SNHG16 was a frequent event in DLBCL. Consistently, up‐regulated expression of SNHG16 was detected in DLBCL cells compared with normal B lymphocytes. SNHG16 is overexpressed in various types of human cancers.^19^, ^20^, ^21^, ^36^ Moreover, increased expression of SNHG16 independently predicts a poor prognosis of patients with NSCLC^22^ and bladder cancer.^20^ In addition, serum SNHG16 expression is suggested as a potential early diagnostic biomarker for lung cancer.^37^ Here we found that DLBCL tissues from patients with advanced tumour stages showed prominent higher levels of SNHG16 compared with those from patients with early tumour stages. Several studies have revealed the mechanism involved in the deregulation of SNHG16 in human cancer. In CRC cells, inactivation of Wnt signalling leads to decreased expression of SNHG16.^19^ The proto‐oncogene c‐Myc is identified as an upstream regulator of SNHG16 in OSCC.^36^ Moreover, upstream transcription factor 1 (USF1) positively regulates the expression of SNHG16 by directly binding to the promoter region of SNHG16 in glioma cells.^38^ Therefore, the collection of more DLBCL samples is merited to investigate the diagnostic and prognostic potential of SNHG16, as well as to further explore the potential mechanism underlying the overexpression of SNHG16 in DLBCL.

Recent studies have demonstrated that lncRNAs function as oncogenes or tumour suppressors in DLBCL. For instance, lncRNA SMAD5‐AS1 plays a tumour suppressive role in DLBCL by inhibiting cell proliferation in vitro and repressing tumour growth in vivo.^39^ LncRNA PANDA leads to cell cycle arrest at G0/G1 phase in DLBCL cells.^40^ Conversely, lncRNA MALAT‐1 contributes to proliferation and cell cycle progression, and it represses apoptosis of DLBCL cells.^41^ Additionally, lncRNA HULC has a tumour‐promoting role in DLBCL.^35^ In this study, SNHG16 knockdown‐induced inhibition of proliferation, G0/G1 phase arrest and apoptosis of OCI‐LY3 and OCI‐LY7 cells, and it suppressed in vivo tumour growth of OCI‐LY7 cells. These results suggested that SNHG16 could facilitate DLBCL progression by regulating cell proliferation, cell cycle progression and apoptosis. However, an artificial model was employed in this study, thus providing only an idea about the underlying mechanism and necessitating further studies. SNHG16 has been identified as a ceRNA in other tumour models.^21^, ^38^, ^42^ Thus, we further investigated the candidate target miRNAs for SNHG16 in DLBCL based on an online platform. The luciferase reporter assay in HEK293T cells and RNA pull‐down assay in DLBCL cells demonstrated that SNHG16 acted as a ceRNA by directly interacting with miR‐497‐5p and inversely regulated its abundance in DLBCL cells. miR‐497‐5p was underexpressed and negatively correlated with SNHG16 expression in DLBCL tissues. A previous study has found that low expression of miR‐497‐5p indicates poor clinical outcomes in DLBCL patients,^28^ which is consistent with the tumour suppressive potential of miR‐497‐5p in our study. In addition, the luciferase reporter assay in HEK293T cells indicated that miR‐497‐5p functioned as a post‐transcriptional regulator of PIM1 by directly binding to the 3′UTR. PIM1 was highly expressed in DLBCL and inversely correlated with miR‐497‐5p expression. A previous study has demonstrated that overexpression of PIM1 is correlated with poor clinical outcomes of DLBCL patients.^33^ Functionally, PIM1 contributes to cell proliferation in vitro and tumour growth in vivo, indicating a potential therapeutic target in DLBCL.^33^, ^43^ Interestingly, SNHG16 overexpression rescued miR‐497‐5p‐induced repression of PIM1. Accordingly, the restoration of PIM1 expression abolished SNHG16 knockdown‐induced cell proliferation inhibition, cell cycle arrest and apoptosis of DLBCL cells. Thus, our data revealed that SNHG16 facilitated tumour growth of DLBCL by targeting the miR‐497‐5p/PIM1 axis.

In summary, these results provide new insights into the tumour‐promoting role of the SNHG16/miR‐497‐5p/PIM1 axis in DLBCL progression, which may improve our understanding of the pathogenesis of DLBCL and provide potential therapeutic targets.

## 5 CONCLUSIONS

To conclude, we have found that lncRNA SNHG16 is highly expressed in DLBCL. Knockdown of SNHG16 inhibits DLBCL cell proliferation, suppresses cell cycle progression and induces apoptosis in vitro and in vivo. Mechanistically, SNHG16 functions as a ceRNA by directly interacting with miR‐497‐5p. PIM1 is a novel downstream target of miR‐497‐5p and mediates the tumour‐promoting role of the SNHG16/miR‐497‐5p axis in DLBCL cells. Our results support the notion that the SNHG16/miR‐497‐5p/PIM1 axis may provide novel potential targets for DLBCL therapy.

## CONFLICT OF INTEREST

All authors declare no conflicts of interest.

## AUTHOR CONTRIBUTIONS

Qiuran Xu and Xiangmin Tong conceived and designed the experiments; Qiaojuan Zhu, Yazhao Li, Yang Guo, Linjun Hu, Zunqiang Xiao, and Xin Liu performed the experiments; Qiaojuan Zhu, Yazhao Li and Yang Guo analysed the data; Jiahui Wang contributed reagents/materials/analysis tools; Qiaojuan Zhu and Qiuran Xu wrote the paper. All authors read and approved the final manuscript.

## Data Availability

The data that support the findings of this study are available from the corresponding author upon reasonable request.
