# Supplementary figures and images for: Long non‐coding RNA SNHG16 promotes proliferation and inhibits apoptosis of diffuse large B‐cell lymphoma cells by targeting miR‐497‐5p/PIM1 axis
Source: J Cell Mol Med. 2019 Sep 4;23(11):7395–405. doi: 10.1111/jcmm.14601 (PMC6815839; doi:10.1111/jcmm.14601)

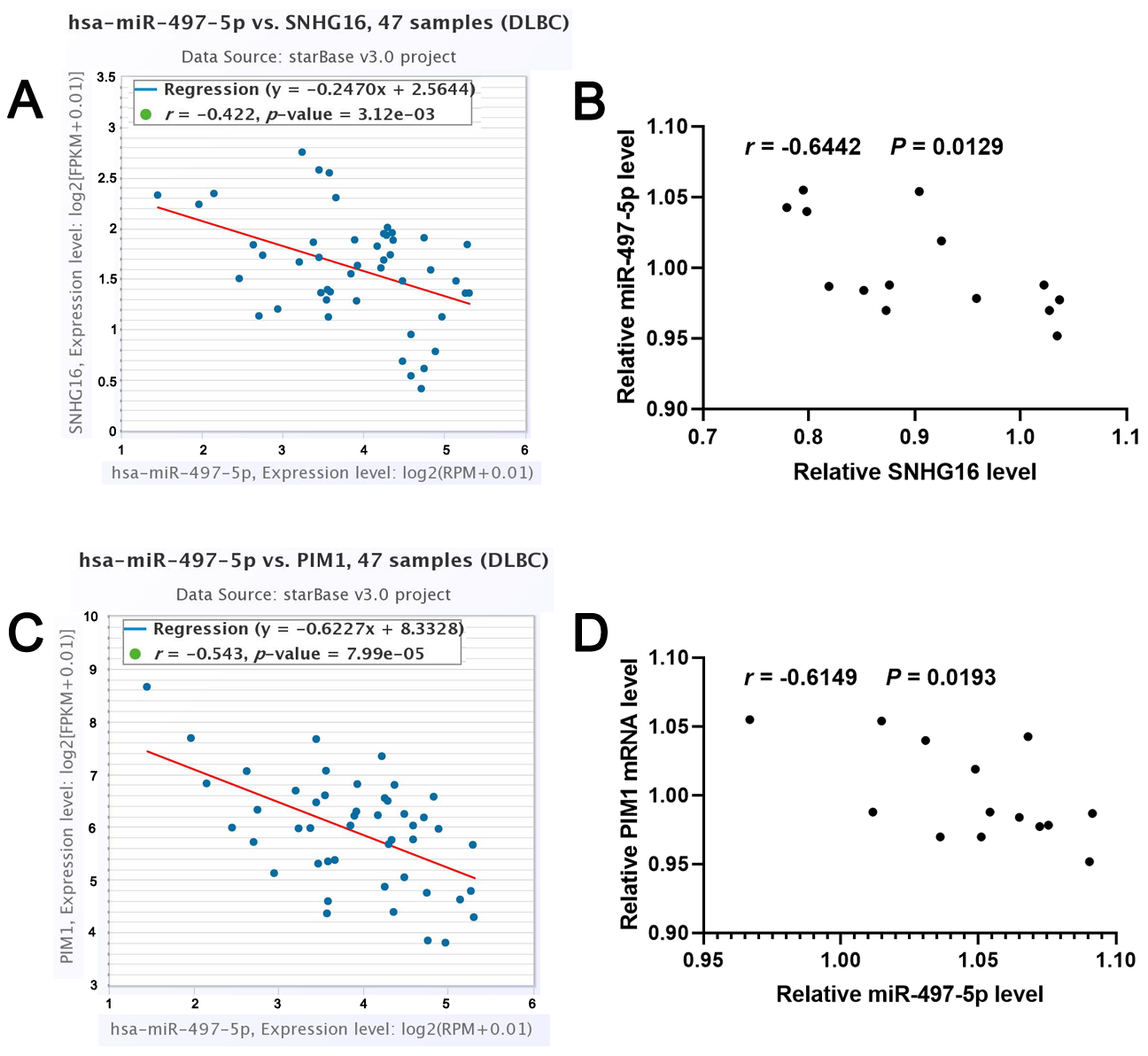

Supplement: Supplementary file 1 [file JCMM-23-7395-s001.tif]
